# Non-healing painful ulcers in a patient with chronic kidney disease and role of sodium thiosulfate: a case report

**DOI:** 10.1186/1757-1626-1-178

**Published:** 2008-09-23

**Authors:** Arshdeep Tindni, Kumar Gaurav, Mukta Panda

**Affiliations:** 1 Department of Medicine, University of Tennessee, College of Medicine, Chattanooga, TN, USA

## Abstract

**Introduction:**

Calciphylaxis is a rare but dreadful complication of chronic kidney disease. It is characterized by nodular subcutaneous calcification and painful tissue necrosis which often results in ulceration and secondary infection, leading to a high rate of mortality. Pathogenesis of this disease is not well understood and there are very few, poorly studied treatment options for this disease.

**Case presentation:**

We report a case of 60 year old Caucasian female with history of long standing diabetes and chronic kidney disease stage 5 who presented with a very high calcium phosphorous product, markedly elevated intact PTH levels and X-ray changes consistent with diagnosis of calciphylaxis. Patient was initially managed by parathyroidectomy and later on was also treated with intravenous sodium thiosulfate thrice weekly with each hemodialysis session for duration of 10 weeks.

**Conclusion:**

Patient had rapid and dramatic relief of her symptoms of pain. Treatment with sodium thiosulfate was well tolerated without any side effects, accompanied by significant symptomatic relief but without any effect on disease progression. So the role of sodium thiosulfate in calciphylaxis is unknown and need further studies.

## Introduction

Calciphylaxis is an inadequately understood syndrome of vascular calcification and skin necrosis affecting around 1% of patients with end stage renal disease (ESRD) [1]. It is thought to result from multiple comorbid factors. This disease carries a very high morbidity and an extremely high mortality rate. Young patients with prolonged duration of renal replacement therapy are the most commonly affected patient population [2]. There are very few, poorly studied treatment modalities reported in literature. We report a case of a 60 year old female with a history of long standing diabetes and chronic kidney disease that presented with painful skin lesions and was diagnosed with calciphylaxis. In our patient, due to the lack of response to more commonly described treatment strategies we utilized the newly described but less studied treatment modality of sodium thiosulfate which has reportedly shown marked improvement in symptom control in literature [[3,4], and [5]].

## Case report

**Figure 1:**
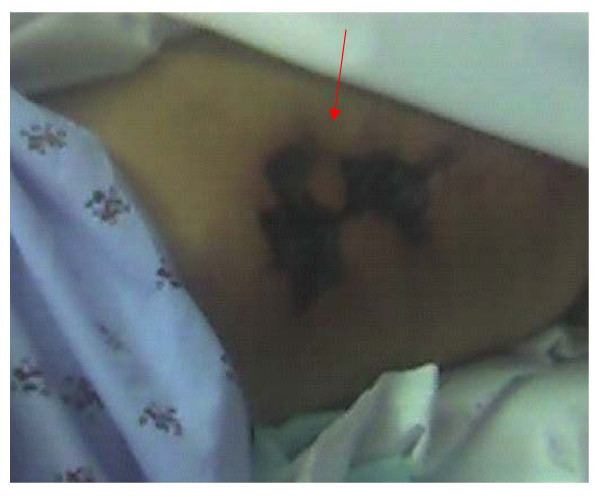
Picture of left thigh at fifth day of hospitalization.

**Figure 2:**
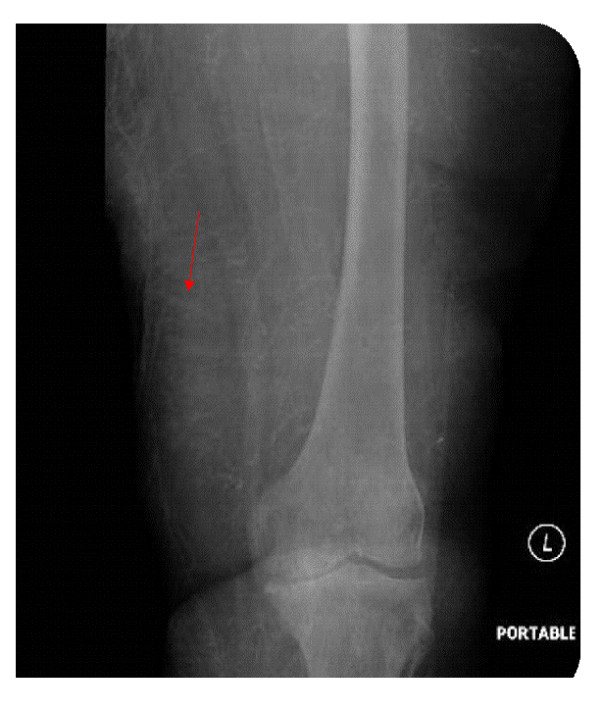
X-ray of left thigh (AP view) showing extensive vascular Calcification.

**Figure 3:**
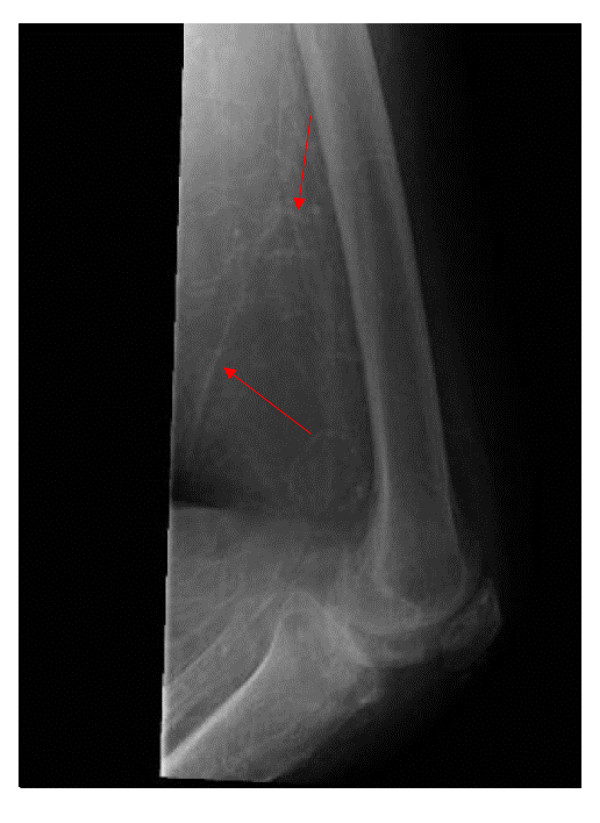
X-ray left thigh (lateral view) showing extensive vascular Calcification.

A 60-year-old Caucasian woman with a history of stage 5 chronic kidney disease presented with complaints of heaviness and pain in the medial aspect of left thigh, worsening over seven days. She denied any fever, chills or rigors. Past medical history was remarkable for coronary artery disease, hypertension, well controlled Type II diabetes mellitus diagnosed nine years ago, (hemoglobin A_1_C of 5.6, six months prior to presentation) and chronic kidney disease stage 5 (glomerular filtration rate: 12 ml/min/1.73 m^2^). Her medications included pioglitazone, furosemide, atenolol, lovastatin, lisinopril, isosorbide mononitrate and metolazone. Physical exam revealed some tenderness on the left thigh region but no edema or erythema. She had palpable pulses bilaterally. Rest of the physical exam was within normal limits. A doppler ultrasound performed on bilateral lower extremities was negative for deep vein thrombosis. Laboratory data revealed a white blood cell count 13.1 thousand/mm3, hemoglobin 8.6 mg/dl, blood urea nitrogen 52 mmol/l, creatinine 4.7 mmol/l (baseline creatinine around 5.0 mmol/l), calcium 10.4 mmol/l, albumin 2.2 gm/dl, phosphorus 8 mmol/l and a parathyroid hormone (PTH) level of 2138 pg/dl (with a level of 1381 pg/dL 6 months prior) On the fifth day of hospitalization, she started developing peridermal necrotic areas along with erythema and tenderness in her thigh (Figure 1). She was initially started on vancomycin as the lesions were suspected to be secondary to local cellulitis. However the absence of fever and the presence of a markedly raised PTH levels along with the clinical picture of widespread necrosis with erythema made calciphylaxis a possibility. X-ray of left thigh was then obtained demonstrating extensive vascular calcifications (figures 2 and Figure 3) thus making calciphylaxis highly likely on our list of differential diagnosis. We did not perform biopsy of the plaques as biopsy of a calciphylactic tissue is known to precipitate ulceration and subsequent infection [6,7]. Patient was started on chronic renal replacement therapy and pain control and discharged home to be followed up as an outpatient. She presented back to the hospital because of worsening left thigh ulceration and related pain. Subtotal parathyroidectomy was then performed on this hospitalization because of very high concentration of parathyroid hormone. After parathyoidectomy, patient's calcium phosphorus product decreased from 80 to 30. Serum calcium and phosphorus had decreased to 8.2 mg/dl and 3.7 mg/dl respectively. On out patient follow up her serum intact parathyroid hormone level decreased to19 pg/ml over a 2 month period. Her skin lesions continued to progress and she complained of significant pain related to these lesions. We then performed a thorough literature review regarding various treatment options available for calciphylaxis and had detailed discussion with the patient regarding the same. Decision was made to try sodium thiosulfate. She was administered sodium thiosulfate therapy 3 times weekly (25 g intravenously over 30 to 60 minutes) following each hemodialysis session. Over the next 2 weeks patient had significant improvement in her pain symptoms and marked decrease in analgesic demand. She was then transferred to a nursing home with continued use of sodium thiosulfate along with each hemodialysis session. After 3 weeks patient was readmitted to the hospital with worsening skin lesions extending to involve her abdomen, buttocks and calf region in bilateral lower extremities. Patient was continued on sodium thiosulfate for 4 more weeks but her skin lesions continued to progress and deteriorate. Various other management options were again discussed with the patient and the decision was reached to keep her comfortable and her management was transferred to hospice care

## Discussion

Calciphylaxis or calcific uremic arteriolopathy is an extraosseous calcification of soft tissue and blood vessels and is characterized by systemic medial calcification of the arteries that leads to ischemia and skin and soft tissue necrosis [8]. It is an uncommon but grave complication in adult patients with chronic kidney disease (CKD) and end stage renal disease. Calciphylaxis occurs in about 1% of End stage renal disease patients each year [1]. It has a very high 5 year mortality rate approaching as high as 65% [9]. The classic presentation may start with tender violaceous or mottled skin lesions, plaques and/or subcutaneous nodules which progress to ischemic non-healing deep ulcers with subcutaneous fat necrosis. These may be complicated by infection.

The pathogenesis of calciphylaxis is very poorly understood hence making the treatment of this rare but frequently fatal disease quite difficult. It is probably multifactorial in etiology. It is more common in Caucasian females. Commonly associated conditions in the pathogenesis of calciphylaxis include obesity, hyperphosphatemia, an elevated calcium-phosphate product, secondary hyperparathyroidism, high alkaline phosphatase and low serum albumin [9]. The most interesting fact is that although these abnormalities are quite common in patients with End stage renal disease, calciphylaxis is rather uncommon. Various hypotheses have been postulated regarding the pathogenesis of this disease including vascular calcium deposition leading to tissue ischemia [10]. This hypothesis has been supported by the fact that the use of non-calcium based phosphate binders and hemodialysis with low calcium dialysate has demonstrated some benefit in some patients [11].

Diagnosis of this condition may sometimes be difficult and requires a high index of clinical suspicion, particularly in patients with renal impairment. Patients may have high levels of PTH, calcium, phosphorous, calcium phosphorous product, alkaline phosphatase, urea and creatinine value however calciphylaxis may occur with normal calcium and phosphate levels. Traditional radiographic studies may demonstrate a 'pipe-stem' pattern of vascular calcification. Also, xeroradiography may demonstrate calcification of subcutaneous arterioles [12]. Bone scan is another radio diagnostic study which may help in diagnosis and also to monitor disease progression Biopsy of the lesions, which is considered gold standard, may reveal calcification and endovascular fibrosis of small subcutaneous arteries and arterioles, which is sometimes associated with infarction of the skin.

There are multiple differential diagnoses to be considered for this condition including vasculitic syndromes, cholesterol emboli syndrome, cryoglobulinemia, cryofibrinogenemia, warfarin induced skin necrosis, disseminated intravascular coagulation (DIC), nephrogenic fibrosing dermopathy (NFD), scleromyxedema, type 1 primary oxaluria, livedoid erythema, antiphospholipid antibody syndrome, fungal infections, panniculitis, pyoderma gangrenosum, atherosclerotic peripheral vascular disease, cellulitis and necrotizing fasciitis. Thus the diagnostic work up in for this condition in doubtful cases may include multiple diagnostic tests for the above mentioned conditions based on clinical suspicions.

There are various treatment modalities described in literature which includes dietary modifications, use of non calcium phosphorous binders and low calcium bath dialysis. Calcimimetics and parathyriodectomy may be beneficial in patients with hyperparathyroidism [13,14]. Some case reports have discussed the use of hyperbaric oxygen. On our literature review we did not come across any prospective controlled studies comparing various treatment modalities. Recently, over the last few years, one of the proposed therapies involves the use of sodium thiosulfates which increases the solubility of calcium deposits [15]. Successful use of this agent has been reported for managing nephrolithiasis [15] and tumoral calcinosis [8]. The success of sodium thiosulfate therapy in patients with calciphylaxis has been described in several case reports and case series with improvement in symptoms and healing of the ulcers [[3,4], and [5]].

Sodium thiosulfate pentahydrate (Na^2^S^2^O^3^) has a molecular weight of 248 (KDa) and has a neutral pH in water. It distributes throughout extracellular fluids and is excreted unchanged in the urine, as is described in normal animals. It has been classically used as an antidote for acute cyanide poisoning and as a topical agent in treatment for acne and pityriasis versicolor. Sodium thiosulfate reacts with calcium resulting in a calcium thiosulfate salt of extremely high soluability [15]. Calcium thiosulfate is 3600 times more soluble than calcium phosphate [15]. In patients with ESRD on hemodialysis sodium thiosulfate is probably eliminated via biliary secretion.

Our patient was diagnosed with calciphylaxis based on the overall clinical picture including symptoms, signs, progression of the lesions and characteristic radiographic findings. Although biopsy is considered gold standard for diagnosis it was not performed in our patient given the high likelihood of clinical suspicion and the fear of causing ulceration and infection. Our patient had debilitating calciphylactic lesions with very high intact PTH levels. Initial unsuccessful trial of renal replacement therapy was done without much clinical benefit to the patient. Thereafter parathyroidectomy was performed with the thought process that it decreases the calcium phosphorous product but again was clinically unsuccessful. Later on she was also treated with sodium thiosulfate with the rationale that it may solubilize the calcium deposits. Our patient did have the benefit of drastic pain relief but unfortunately the progression of disease and skin lesions was unaffected, unlike the cases described in the literature. The reason for this may be related to the rarity of reporting of this condition and paucity of comparative studies or maybe a reflection of the relative low reporting of negative outcomes.

## Conclusion

Calciphylaxis persists to be a dreadful condition, with poorly understood pathophysiology. The ideal treatment of this disease is unknown because of its rarity and the lack of prospective clinical trials. Sodium thiosulfate seems to be of some clinical benefit in adult patients with calciphylaxis. It is well tolerated and has minimal adverse effects. Further prospective studies are needed to establish the role of sodium thiosufate in the treatment of this entity. We believe that though our patient did not have an effect on disease progression she had rapid improvement in her symptoms following administration of sodium thiosulfate.

## Consent

The patient is now deceased. Written informed consent for publication of this manuscript and any accompanying images was obtained from the patient's daughter. A copy of the written approval is available for review by the Editor-in-Chief of this Journal.

## Competing interests

The authors declare that they have no competing interests.

## Authors' contributions

AT participated in patient management, literature review and helped in conceptualization of manuscript. KG and MP helped in conceptualization, reviewing and drafting of the manuscript. All authors read and approved the final manuscript.
